# Sulfated Hetero-Polysaccharides Protect SH-SY5Y Cells from H_2_O_2_-Induced Apoptosis by Affecting the PI3K/Akt Signaling Pathway

**DOI:** 10.3390/md15040110

**Published:** 2017-04-06

**Authors:** Jing Wang, Huaide Liu, Xuan Zhang, Xinpeng Li, Lihua Geng, Hong Zhang, Quanbin Zhang

**Affiliations:** 1 Key Laboratory of Experimental Marine Biology, Institute of Oceanology, Chinese Academy of Sciences, Qingdao 266071, China; Jingwang@qdio.ac.cn (J.W.); xinpengli@163.com (X.L.); lihuageng@163.com (L.G.); iocwang@ucdavis.edu (H.Z.); 2 School of Life Sciences, Nantong University, Seyuan Road 9, Nantong 226019, China; hold126@126.com; 3 Taian City Central Hospital, Taian 271000, China; sdnyzhangxuan@163.com; 4 Laboratory for Marine Biology and Biotechnology, Qingdao National Laboratory for Marine Science and Technology, Qingdao 266071, China

**Keywords:** sulfated hetero-polysaccharides, PI3K/Akt, phosphorylate, SH-SY5Y, oxidative stress

## Abstract

Parkinson’s disease (PD) is one of the most common neurodegenerative diseases. Recent studies suggest that sulfated hetero-polysaccharides (UF) protect against developing PD. However, the detailed mechanisms of how UF suppress neuronal death have not been fully elucidated. We investigated the cytoprotective mechanisms of UF using human dopaminergic neuroblastoma SH-SY5Y cells as a PD model. UF prevented H_2_O_2_-induced apoptotic cell death in SH-SY5Y cells in a dose-dependent manner. An examination of the PI3K/Akt upstream pathway revealed that UF-pretreated cells showed a decreased relative density of Akt, PI3K, and TrkA, and increased the phosphorylation of Akt, PI3K, and NGF; the PI3K inhibitor, LY294002, partially prevented this effect. An examination of the PI3K/Akt downstream pathway revealed the increased expression of the apoptosis-associated markers Bax, p53, CytC, and GSK3β, and the decreased expression of Bcl-2 in UF-treated cells. UF-treated cells also exhibited decreased caspase-3, caspase-8, and caspase-9 activities, which induced cell apoptosis. Our results demonstrate that UF affect the PI3K/Akt pathway, as well as downstream signaling. Therefore, the UF-mediated activation of PI3K/Akt could provide a new potential therapeutic strategy for neurodegenerative diseases associated with oxidative injury. These findings contribute to a better understanding of the critical roles of UF in the treatment of PD.

## 1. Introduction

Parkinson’s disease (PD), the second most common neurogegenerative disease in the elderly, is pathologically characterized by the loss of dopaminergic neurons (DA) in the substantia nigra and in the striatum [1]. Research suggests that there are several factors such as apoptosis, oxidative stress, genetic factors, environmental factors, mitochondrial function defect, and ubiquitin proteasome system dysfunction, which may be related to the pathogenesis of PD [2,3,4,5,6]. Neuroprotection is a useful therapeutic strategy for PD; however, there are almost no such drugs in clinic for halting or retarding the degeneration of dopaminergic neurons. Recently, much research has focused on a neuroprotective strategy, and several drugs have been proposed as candidate neuroprotective agents for PD [7,8]. 

The exact reason for the loss of DA neurons is not clear yet; however, numerous studies have shown that the main cause of the loss of DA neurons is apoptosis [9]. Most researchers believe that oxidative stress, a lack of antioxidant function, and mitochondrial function damage could induce the apoptosis of DA neurons on different levels [10]. The mechanism of oxidative stress is the overproduction of reactive oxygen species (ROS), leading to the damage of both neurons and astrocytes [11]. Oxidative stress can cause apoptosis through a series of reactions such as activated caspase, a change in the amount of Bcl-2 and Bcl-2 Associated X protein (Bax), and released Cytochrome c (CytC) [12]. Sufficient evidence has indicated that the down-regulation of the apoptosis protein Bax and the up-regulation of the anti-apoptosis protein Bcl-2 could control the survival of DA neurons [13]. As a result, the regulation of the apoptosis and anti-apoptosis genes and proteins to protect DA neurons has become another PD treatment strategy.

Fucoidan (FPS) is a sulfated polysaccharide extracted from *Saccharina japonica*, with a heterogeneous molecular weight and complex chemical composition. A rpevious study found that FPS exhibited a protective effect in a 1-methyl-4-phenyl-1,2,3,6-tetrahydropyridine (MPTP)-induced neurotoxicity model via its antioxidative activity [14]. Because of its activity, FPS can be used in the drug discovery area. FPS can be purified into several fractions, ranging from high-uronic-acid, low-sulfate, and fucose-containing polymers, to highly sulfated fucoidan. Our preliminary study showed that various fractions of FPS differing in uronic acid and sulfate content showed variable activities [15]. Recent studies have verified that UF have the most complex chemical composition and show the highest neuroprotective activity in vitro and in vivo. The neuroprotection induced by UF may be partly related to their antioxidative activity and anti-apoptotic ability [16]. However, the mechanism of UF in the protection of dopaminergic neurons remains unknown.

In this study, we used an oxidative stress model evoked by hydrogen peroxide (H_2_O_2_). H_2_O_2_ is commonly used to induce apoptosis in dopaminergic cells, including human SH-SY5Y cell lines [17]. Recently, certain members have been shown to play important roles in neuronal apoptosis in response to environmental stresses and apoptotic agents [18]. The Phosphatidylinositol 3-kinase (PI3K)/A serine/threonine kinase (Akt) signaling pathway plays an important role in neuronal survival and death. The anti-apoptotic effects of PI3K are mediated by its downstream target Akt, which can regulate the expression of several apoptosis-related genes, such as Bcl-2/Bax [19]. The up-regulation of Bcl-2 expression is a critical mechanism for cell survival. Nerve growth factor (NGF)/Tropomyosin receptor kinase A (TrkA) signaling can prevent apoptosis via the activation of the PI3K/Akt pathway. However, the effects of UF on PI3K/Akt in a neuronal cell line remain unknown.

The objective of the present study was to study the mechanism of UF on PD disease through the PI3K/Akt signal channel in vitro. We wonder whether UF exhibits anti-DA neuron apoptosis activity through the active PI3K/Akt signal channel. Furthermore, we will study the expression of genes and proteins such as the Blc-2 family and caspase family, in order to confirm that the PI3K/Akt signal channel has a relationship with the protection ability of UF on DA. The results of the research not only supply new ideas and methods for anti-PD mechanism studies of the sulfated heteropolysaccharide, but also provide a scientific basis for the anti-PD drug development.

## 2. Results

### 2.1. Chemical Analysis

UF was prepared from low molecular weight fucoidan (DF), which was fractionated by stepwise elution from DEAE-Sepharose FF using increasing concentrations of aqueous NaCl. UF was obtained from column chromatography with 0.5 M NaCl. The yield of the UF fraction was 11.53%. The chemical composition of the DF and UF fractions are shown in Table 1. The main chemical components of DF and UF were fucose and sulfate, along with uronic acid and a small amount of other monosaccharides. The fucose content of UF and DF was 19.12% and 28.71%, respectively. The sulfate content of UF was lower than that of DF; however, UF had a higher uronic acid content. Mabeau et al. found that fucoidan could be purified into several fractions, ranging from high-uronic-acid, low-sulfate, and fucose-containing polymers, to highly sulfated fucoidans [20]. This finding agrees with our results. The constituents of the neutral monosaccharides of DF and UF were analyzed by HPLC. Fucose was the principal sugar unit in all samples. Galactose was the second most abundant sugar, especially in DF and UF, with molar ratios of 0.579 and 0.713, respectively. In addition to fucose and galactose, other monosaccharides were also observed in the samples, including mannose, glucose, rhamnose, and arabinose. The molecular weight of the polysaccharides is the average molecular. The peak of DF is wider than its fractions and the molecular range is from about 4000 to 11,000 Da, with an average molecular weight of 9544 Da. However, the peak and the molecular range of UF are narrow and the average weight is 6500 Da.

The structure of UF is very complex, which was previously published by our group. Briefly, UF has a backbone of alternating 4-linked GlcA and 2-linked Man, with the first Man residue from the non-reducing end, accidentally sulfated at C6. In addition, UF has a 3-linked glucuronan, in accordance with a previous report by NMR. Some other structural characteristics include GlcA 1→3 Man 1→4 GlcA, Man 1→3 GlcA 1→4 GlcA, Fuc 1→4 GlcA, and Fuc 1→3 Fuc. Finally, fucose is sulfated at C2 or C4, while galactose is sulfated at C2, C4, or C6 [21].

### 2.2. Protective Effect of UF on H_2_O_2_-Induced Neurotoxicity in SH-SY5Y Cells

**Figure 1:**
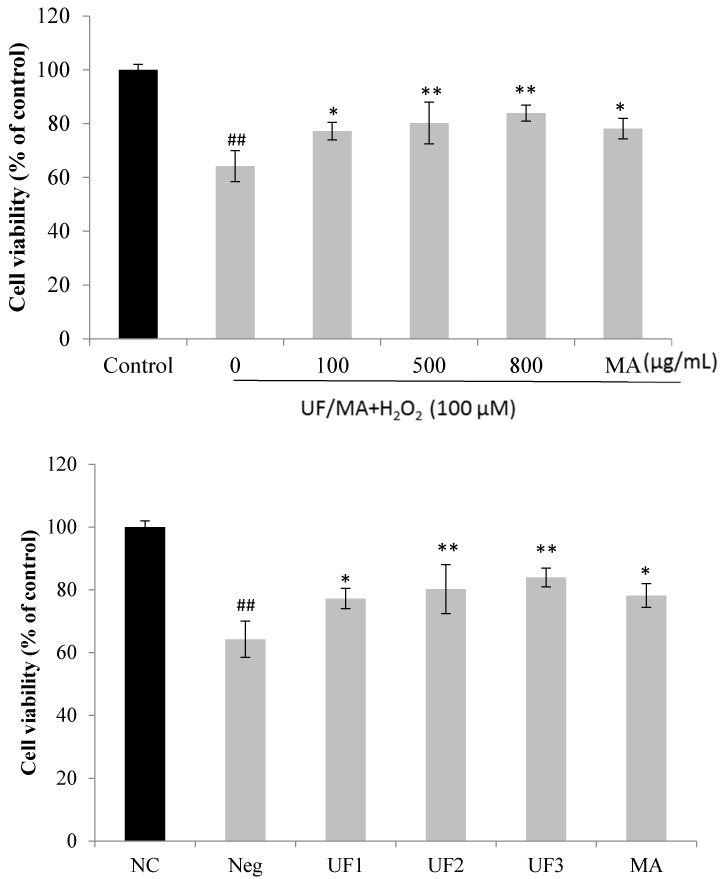
Protective effects of UF on H_2_O_2_-induced cell death. NC: Normal Control group, Neg: Negtive Control group, UF1: UF 100 µg/mL group, UF2: UF 500 µg/mL group, UF3: UF 800 µg/mL group, MA: Positive Control group, * *p* < 0.05; ** *p* < 0.01 (vs. Neg); ^##^ *p* < 0.01 (vs. NC).

Figure 1 summarizes the effect of the samples on the neuronal injury induced by H_2_O_2_ in vitro. In the experiment, the exposure of the SH-SY5Y cells to different concentrations of UF samples had nearly no effect on cell viability. However, the exposure of the SH-SY5Y cells to H_2_O_2_ significantly reduced cell viability; 100 µM and 400 µM H_2_O_2_ decreased cell viability to 60% compared with that of normal cells. In contrast, the administration of samples reversed the decreased cell viability induced by H_2_O_2_ (*p* < 0.01 or *p* < 0.001). UF exhibited the best activity at 800 µg/mL. The in vitro results provide further evidence that UF directly protects against neuronal injury caused by H_2_O_2_.

**Figure 2:**
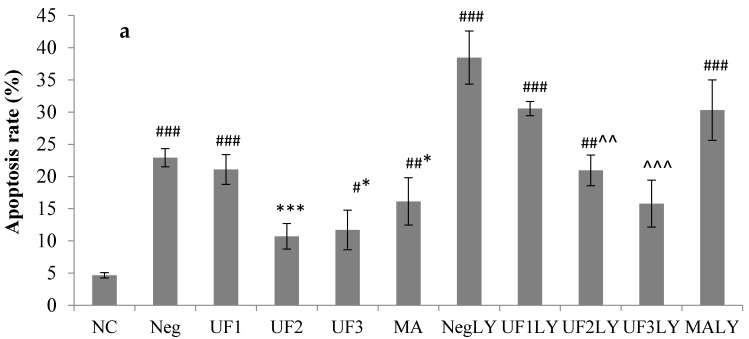

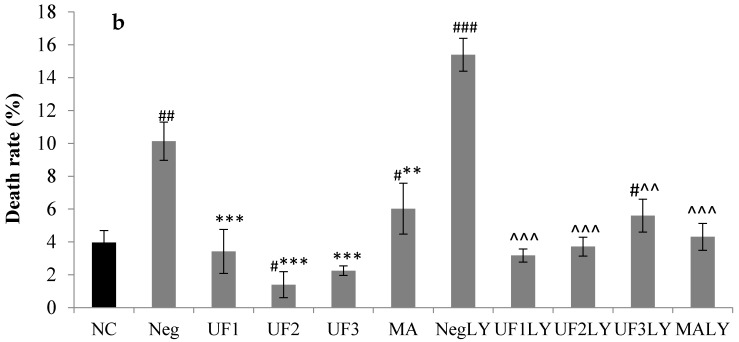
Percentage of apoptotic cells detected in SH-SY5Y cells treated with H_2_O_2_ and UF for 48 h (**a**). Percentage of dead cells detected in SH-SY5Y cells treated with H_2_O_2_ and UF for 48 h (**b**). Data are expressed as percentages and represent the mean ± SD of three separate experiments, in which at least 200 cells were counted per one treatment group. NC: Normal Control group, Neg: Negtive Control group, UF1: UF 100 µg/mL group, UF2: UF 500 µg/mL group, UF3: UF 800 µg/mL group, MA: Positive Control group, NCLY: Normal Control group + LY294002, NegLY: Negtive Control group + LY294002, UF1LY: UF 100 µg/mL group + LY294002, UF2LY: UF 500 µg/mL group + LY294002, UF3LY: UF 800 µg/mL group + LY294002, MALY: Positive Control group + LY294002. ^#^ *p* < 0.05, ^##^ *p* < 0.01, ^###^ *p* < 0.001 (vs. NC), * *p* < 0.05, ** *p* < 0.01, *** *p* < 0.001 (vs. Neg), ^^ *p* < 0.01, ^^^ *p* < 0.001, (vs. NegLY).

To determine whether the samples could affect apoptosis in SH-SY5Y cells, cells were stained with the DNA dye Hoechst 33342 to visualize the nuclear morphology (Figure 2 and Figure S2). Dead cells were stained with the DNA dye propidium iodide (PI). Apoptotic and dead cells were assayed under a microscope. In the absence of H_2_O_2_, the cultured neurons exhibited normal cellular morphology with extending neuritis and evenly attained nuclei. H_2_O_2_ caused morphological changes characteristic of apoptosis, including the degeneration of neuritis and the shrinkage of cell bodies, as well as the fragmentation and condensation of nuclei. The UF sample could decrease the number of apoptotic and dead cells at 500 µg/mL and 100 µg/mL, respectively (*p* < 0.01). To determine whether the activation of the PI3K pathway contributes to UF protection against H_2_O_2_, we used LY294002, a PI3K inhibitor, to block PI3K activation by UF [22]. Then, we examined the effect of the blocked activation on UF neuroprotection against H_2_O_2_. SH-SY5Y cells were pretreated with 20 µM LY294002, different doses of UF, or both, and were then challenged in the presence of 100 µM H_2_O_2_. In the absence of UF, the LY294002 and H_2_O_2_ treatment group increased basal apoptosis from 4.9% to 38.5%, suggesting that the PI3K pathway may be required for serum-promoted SH-SY5Y cells survival [23]. H_2_O_2_-induced apoptosis occurred in 22.9% of the cell population; the addition of LY294002 increased the frequency of apoptosis to 38.5%. UF at 500 µg/mL greatly reduced H_2_O_2_-induced apoptosis to 10.7%. With the addition of LY294002, the effect of UF at 800 µg/mL on H_2_O_2_-induced apoptosis was 15.8%. There was a large difference in the extent of H_2_O_2_-induced apoptosis when cells were pretreated with LY294002 alone or together with UF (38.5% versus 15.8%, *p* < 0.001). These data suggest that the activation of the PI3-kinase pathway is at least part of the mechanism through which UF protect against H_2_O_2_.

### 2.3. Immunocytochemistry

**Figure 3:**
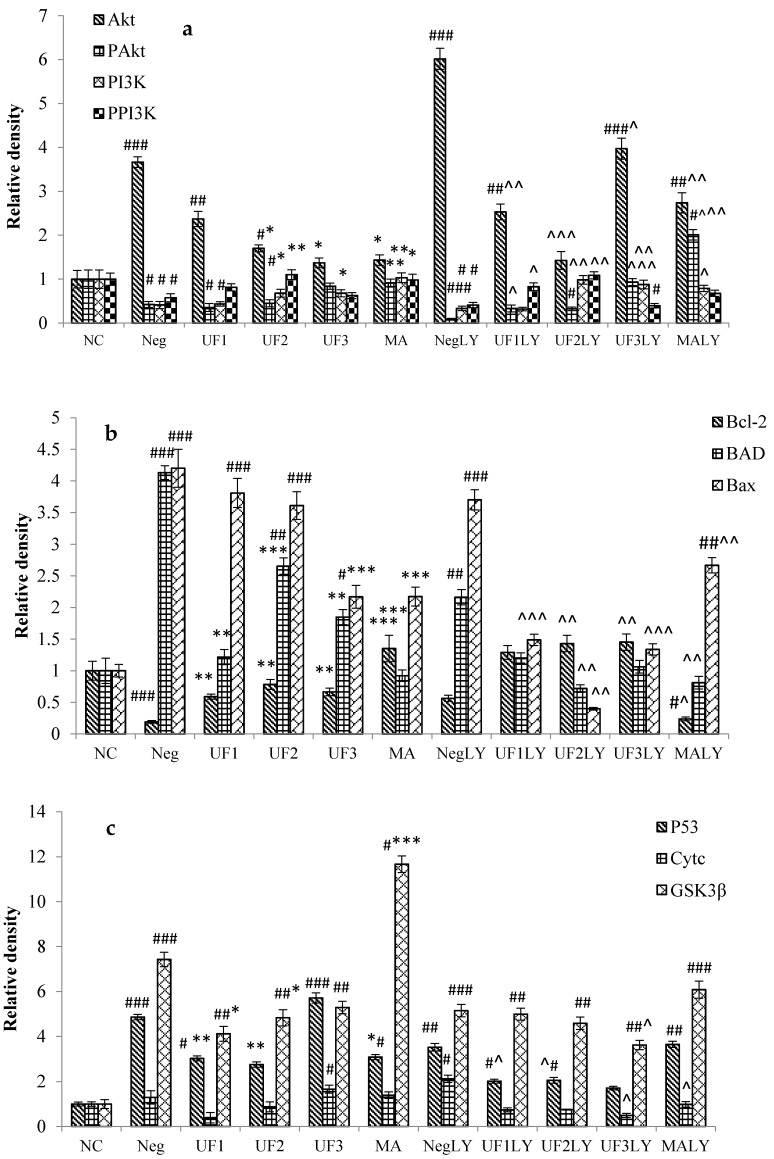

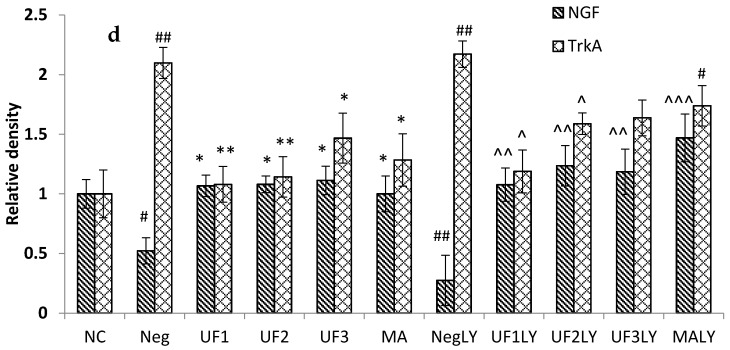
Protective effects of UF on H_2_O_2_-induced SH-SY5Y cells of a relative density of protein in the Akt/PI3K pathway. (**a**). A relative density of protein Akt, PAkt, PI3K and PPI3K; (**b**). A relative density of protein Bcl-2, BAD and Bax; (**c**). A relative density of protein P53, Cytc and GSK3β; (**d**). A relative density of protein NGF and TrkA. NC: Normal Control group, Neg: Negtive Control group, UF1: UF 100 μg/mL group, UF2: UF 500 μg/mL group, UF3: UF 800 μg/mL group, MA: Positive Control group, NCLY: Normal Control group + LY294002, NegLY: Negtive Control group + LY294002, UF1LY: UF 100 μg/mL group + LY294002, UF2LY: UF 500 μg/mL group + LY294002, UF3LY: UF 800 μg/mL group + LY294002, MALY: Positive Control group + LY294002, ^#^ *p* < 0.05, ^##^ *p* < 0.01, ^###^ *p* < 0.001 (vs. NC), * *p* < 0.05, ** *p* < 0.01, *** *p* < 0.001 (vs. Neg), ^ *p* < 0.05, ^^ *p* < 0.01, ^^^ *p* < 0.001, (vs. NegLY).

The levels of some proteins following H_2_O_2_ and UF treatment were examined by immunocytochemistry, in order to determine whether H_2_O_2_ induced SH-SY5Y human neuroblastoma cell death by changing the expression of these proteins. The PD model induced by H_2_O_2_ increased the levels of the apoptosis-promoting protein BAD/Bax and decreased the levels of anti-apoptosis proteins, such as Bcl-2, p53, CytC, and Glycogen synthase kinase 3 beta (GSK3β), but there was a marked decrease in apoptosis-promoting protein levels and an increase in anti-apoptosis protein levels in the UF-treated groups. The ratio of the Bax/Bcl-2 protein can determine the susceptibility of the cell to apoptosis [19]. Consequently, we determined whether cell death occurred by altering the ratio between Bax and Bcl-2. As shown in Figure 3, the treatment of cells with H_2_O_2_ significantly decreased the expression level of Bcl-2 and increased the expression levels of Bax, p53, CytC, and GSK3β. The cells treated with UF had an increased expression level of Bcl-2 and decreased expression levels of Bax and p53 compared with those of the control group. These data suggest that H_2_O_2_ can induce the apoptosis of SH-SY5Y cells and that UF treatment can protect SH-SY5Y cells by regulating the Bax/Bcl-2 ratio.

**Figure 4:**
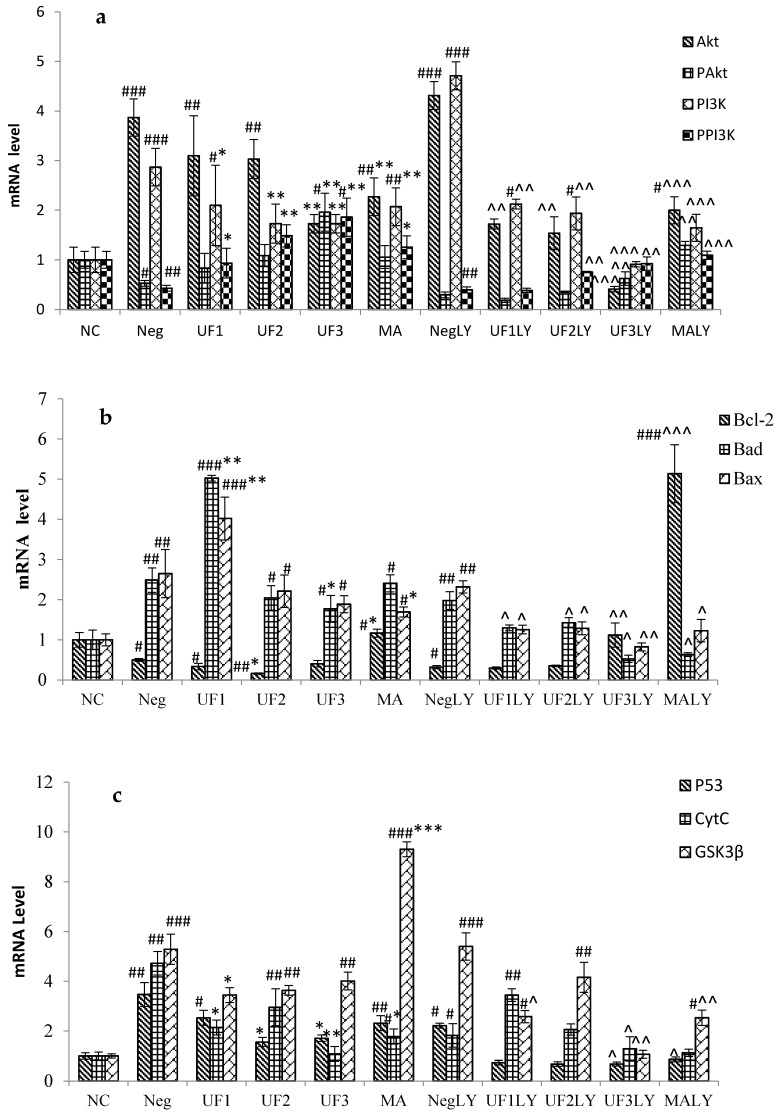

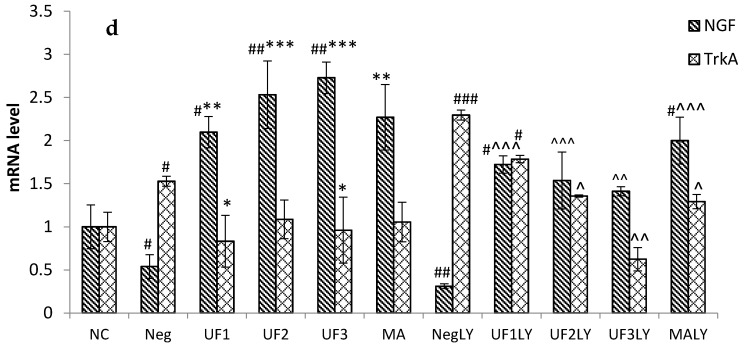
Protective effects of UF on H_2_O_2_-induced SH-SY5Y cells of an mRNA level. (**a**). mRNA level of *Akt, PAkt, PI3K* and *PPI3K*; (**b**). mRNA level of *Bcl-2, BAD* and *Bax*; (**c**). mRNA level of *P53, Cytc* and *GSK3β*; (**d**). mRNA level of *NGF* and *TrkA*. NC: Normal Control group, Neg: Negtive Control group, UF1: UF 100 μg/mL group, UF2: UF 500 μg/mL group, UF3: UF 800 μg/mL group, MA: Positive Control group, NCLY: Normal Control group + LY294002, NegLY: Negtive Control group + LY294002, UF1LY: UF 100 μg/mL group + LY294002, UF2LY: UF 500 μg/mL group + LY294002, UF3LY: UF 800 μg/mL group + LY294002, MALY: Positive Control group + LY294002, ^#^ *p* < 0.05, ^##^ *p* < 0.01, ^###^ *p* < 0.001 (vs. NC), * *p* < 0.05, ** *p* < 0.01, *** *p* < 0.001 (vs. Neg), ^ *p* < 0.05, ^^ *p* < 0.01, ^^^ *p* < 0.001, (vs. NegLY).

To determine whether UF treatment promotes the survival of Congress of Neurological Surgeons (CNS) neurons, we tested the effectiveness of several neurotrophins against H_2_O_2_-induced apoptosis. TrkA levels decreased more in the UF-treated groups than in the H_2_O_2_-treated group (Figure 4). The NGF levels became more elevated in the UF groups (*p* < 0.05, Figure 4b) than in the Model group (Figure 4). The stimulation of PI3K can lead to the activation and phosphorylation of protein kinase Akt [24]. Therefore, we assayed the activity by immunocytochemistry using a phospho-Akt antibody that specifically recognizes activated Akt. The phosphorylation of Akt was detectable in cells maintained under regular culture conditions, probably because of PI3K activation due to the growth factors present in the serum. The addition of UF at 800 µg/mL caused a significant increase in Akt phosphorylation, suggesting that the PI3K pathway is activated by UF.

### 2.4. Real-Time PCR

RT-PCR was used to analyze various amounts of RNA isolated from SH-SY5Y cells exposed to H_2_O_2._ The treatment of cells with 100 μM H_2_O_2_ significantly increased the mRNA level of *Akt* and *PI3K* to 3.87 ± 0.38 and 2.87 ± 0.38 of the control group and decreased that of *PAkt* and *PPI3K* to 0.53 ± 0.06 and 0.43 ± 0.06 of the control group, respectively, (Figure 4; *p* < 0.001, *p* < 0.001, *p* < 0.05, and *p* < 0.01, respectively). However, the mRNA level of *Akt* and *PI3K* significantly decreased to 1.73 ± 0.18 and 1.73 ± 0.22 of the control group, while that of *PAkt* and *PPI3K* significantly increased to 1.96 ± 0.38, 1.86 ± 0.25 of the control group, respectively, when treated with UF at 800 μg/mL (Figure 4; *p* < 0.01, *p* < 0.01, *p* < 0.01, and *p* < 0.01, respectively). Moreover, the treatment of cells with 100 μM H_2_O_2_ and 20 μM LY294002 significantly increased the mRNA level of *Akt* and *PI3K* to 3.87 ± 0.38 and 2.87 ± 0.38 of the control group and decreased that of *PAkt* and *PPI3K* to 0.53 ± 0.06 and 0.43 ± 0.06 of the control group, respectively, (Figure 4; *p* < 0.001, *p* < 0.001, *p* < 0.05, and *p* < 0.01, respectively). However, the mRNA level of *Akt* and *PI3K* significantly decreased to 1.73 ± 0.18 and 1.73 ± 0.22 of the control group and that of *PAkt* and *PPI3K* significantly increased to 1.96 ± 0.38 and 1.86 ± 0.25 of the control group, respectively, when treated with UF at 800 μg/mL (Figure 4; *p* < 0.01, *p* < 0.01, *p* < 0.01, and *p* < 0.01, respectively). We also found that the mRNA expression of the key apoptosis-related genes *Bad*, *Bax*, *p53*, *Cytc*, and *GSK3β* were consistently up-regulated to 2.49 ± 0.30, 2.65 ± 0.60, 3.47 ± 0.48, 4.72 ± 0.47, and 5.29 ± 0.60, respectively, in cells treated with 100 μM H_2_O_2_, whereas that of *Bcl-2* was down-regulated to 0.50 ± 0.04 of the control values. However, UF treatment significantly inhibited the up-regulation or down-regulation of *Bad*, *Bax*, *p53*, *Cytc*, *GSK3β,* and *Bcl-2*. Compared to the H_2_O_2_ group, the UF-treated groups decreased the mRNA levels of *TrkA* and increased the mRNA levels of *NGF*.

### 2.5. Caspase-3, Caspase-8 and Caspase-9 Activity

**Figure 5:**
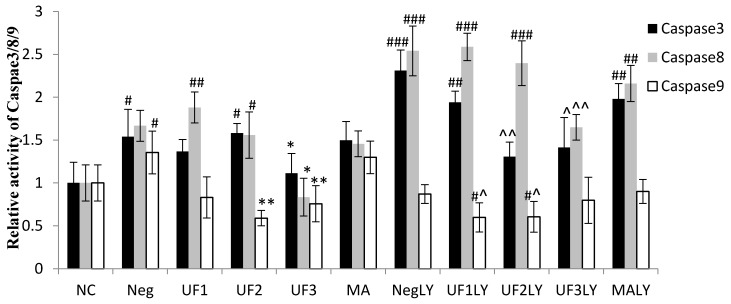
Protective effects of UF on H_2_O_2_-induced SH-SY5Y cells of a relative activity of caspase-3, caspase-8, and caspase-9. NC: Normal Control group, Neg: Negtive Control group, UF1: UF 100 μg/mL group, UF2: UF 500 μg/mL group, UF3: UF 800 μg/mL group, MA: Positive Control group, NCLY: Normal Control group + LY294002, NegLY: Negtive Control group + LY294002, UF1LY: UF 100 μg/mL group + LY294002, UF2LY: UF 500 μg/mL group + LY294002, UF3LY: UF 800 μg/mL group + LY294002, MALY: Positive Control group + LY294002, ^#^ *p* < 0.05, ^##^ *p* < 0.01, ^###^ *p* < 0.001 (vs. NC), * *p* < 0.05, ** *p* < 0.01 (vs. Neg), ^ *p* < 0.05, ^^ *p* < 0.01, (vs. NegLY).

The treatment of cells with 100 μM H_2_O_2_ significantly increased the relative activity of caspase-3, caspase-8, and caspase-9 to 1.54 ± 0.32, 1.67 ± 0.18, and 1.36 ± 0.25 of the control group, respectively (Figure 5; *p* < 0.05, *p* < 0.01, and *p* < 0.01, respectively); however, this significantly decreased to 1.11 ± 0.23, 0.83 ± 0.22, and 0.76 ± 0.21 of the control group, respectively, under UF treatment at 800 μg/mL (Figure 5; *p* < 0.05, *p* < 0.05, and *p* < 0.01, respectively). Moreover, the treatment of cells with 100 μM H_2_O_2_ and 20 μM LY294002 significantly increased the relative activity of caspase-3 and caspase-8 to 3.21 ± 0.24 and 2.54 ± 0.29 of the control group, respectively (Figure 5; *p* < 0.001 and *p* < 0.001, respectively); however, this significantly decreased to 1.41 ± 0.35 and 1.64 ± 0.15 of the control group, respectively, when treated with UF at 800 μg/mL (Figure 5; *p* < 0.01 and *p* < 0.01, respectively). These results suggest that UF treatment could promote the activity of apoptosis-related proteins, such as caspase-3, caspase-8, and caspase-9, in SH-SY5Y cells treated with 100 μM H_2_O_2_ and 20 μM LY294002. 

### 2.6. Western Blotting 

**Figure 6:**
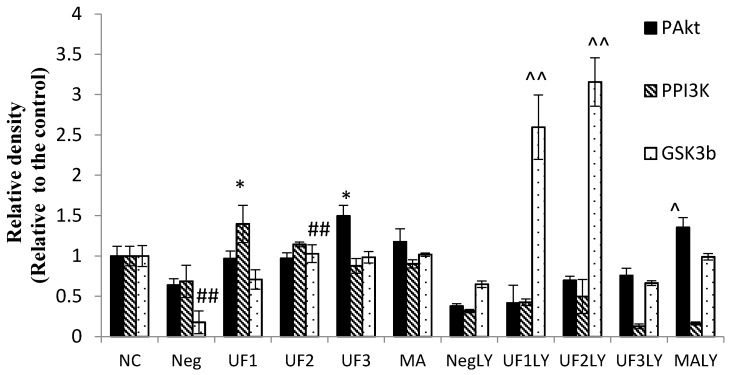
Western blotting analysis of PAkt, PPI3K, and GSK3β proteins on H_2_O_2_-induced SH-SY5Y cells. NC: Normal Control group, Neg: Negtive Control group, UF1: UF 100 μg/mL group, UF2: UF 500 μg/mL group, UF3: UF 800 μg/mL group, MA: Positive Control group, NCLY: Normal Control group + LY294002, NegLY: Negtive Control group + LY294002, UF1LY: UF 100 μg/mL group + LY294002, UF2LY: UF 500 μg/mL group + LY294002, UF3LY: UF 800 μg/mL group + LY294002, MALY: Positive Control group + LY294002, ^##^ *p* < 0.01 (vs. NC), * *p* < 0.05, (vs. Neg), ^ *p* < 0.05, ^^ *p* < 0.01, (vs. NegLY).

The phosphorylation of AKT, PI3K, and GSK3β were also evaluated by western blot. Figure 6 shows that UF3 successfully enhanced the expression of phosphorylated protein PAkt and GSK3β, and UF1 could elevate the expression of phosphorylated protein PPI3K compared to model rats (*p* < 0.05). In addition, after the addition of LY294002, the UF treated group slightly restored the phosphorylated protein expression and significantly enhanced the expression of GSK3β.

## 3. Discussion

PD is associated with the progressive loss of dopaminergic neurons and more widespread neuronal changes that cause complex symptoms. In the previous study, we found that UF directly protected against neuronal injury caused by MPP^+^ and that this neuroprotective effect may be partially mediated through antioxidant activity and the prevention of apoptosis [16]. In this study, we further investigated the possible signaling mechanisms underlying the protective effect of UF on H_2_O_2_-induced cell injury. We focused on PI3K and Akt because both of them are well-known prosurvival protein kinases and are hence thought to be involved in the antioxidation effects that induce neuronal protection [25]. 

Akt, known as protein kinase B (PKB), is a key molecule in growth factor signaling pathways, mediating neuronal survival in both development and disease in multiple paradigms, including resistance against oxidative insults in the brain [26]. Stimulation of the PI3K pathway is usually necessary for Akt activation. Once activated, Akt, in turn, inactivates several pro-apoptotic proteins, including BAD and caspase-9, and thereby promotes cell survival [27]. The current study showed that incubating SH-SY5Y cells with UF increased the PPI3K level. Because the downstream target of PI3K is Akt, we also observed increased phosphorylated Akt expression, suggesting that UF may regulate dopaminergic neuronal survival via PI3K/Akt phosphorylation. We also found that blocking PI3K with the selective inhibitor LY294002 decreased the protective effects of UF on cell viability. More importantly, UF reversed the down-regulated Akt activity caused by-H_2_O_2_. Our data clearly suggest that the protective effect of UF on H_2_O_2_-induced cell injury is mediated by stimulation of the PI3K/Akt pathway. 

The apoptosis executioner caspase-3 is downstream of PI3k/Akt, and its increase is correlated with neuronal death in PD [28]. Signore et al. reported that in primary dopamine neurons, erythropoietin prevented neuronal apoptosis by activating the PI3K/Akt pathway and subsequently decreasing caspase-3 activation [29]. Similarly, in dopaminergic SH-SY5Y cell lines, caspase-3 activation was found to be negatively correlated with Akt phosphorylation [30]. Consistent with Signore’s and Fang’s studies, our result showed that in SH-SY5Y cells, H_2_O_2_ significantly increased the expression of caspase-3, which was accompanied by a decrease in PI3k/Akt phosphorylation. This finding strongly suggests that the PI3K/Akt signaling pathway plays an important role in H_2_O_2_-damaged SH-SY5Y cell death. The up-regulation of caspase-3 and the down-regulation of PI3K/Akt phosphorylation resulting from H_2_O_2_ incubation were reversed after UF treatment. Our results demonstrate that UF mediates neuroprotection in the H_2_O_2_-induced SH-SY5Y model via an anti-apoptotic effect occurring, at least partially, via the PI3K/Akt pathway. To further verify the role of this signaling pathway in UF-mediated neuroprotection, LY294002, an inhibitor of PI3K, was incubated with UF in H_2_O_2_-induced SH-SY5Y cells. We found that the administration of LY294002 with UF led to a partial inhibition of the increased phosphorylation and the increased expression of caspase-3 (Figure 5). Our observation further verifies that UF-mediated neuroprotection in H_2_O_2_-induced SH-SY5Y is at least partially dependent on the PI3K/Akt pathway. The activation of GSK3β results in neuronal PCD and mediates striatal toxin-induced neuronal death, whereas its inhibition promotes neuronal survival. Our results demonstrate that cells with H_2_O_2_ could activate GSK3β and that UF treatment could degrade GSK3β and promote cell survival. 

## 4. Materials and Methods

### 4.1. Preparation of Sulfated Polysaccharides

*Saccharina japonica* (Laminariaceae), cultured along the coast of Rongcheng, China, was collected in August 2015, authenticated by Prof. Lanping Ding and stored as a voucher specimen (No. 83) in the Herbarium of the Algal Chemistry Department, Institute of Oceanology. The fresh algae were promptly washed, sun dried, and kept in plastic bags at room temperature until use. FPS was extracted according to the method of Wang et al., with minor modifications [15]. DF was prepared using ascorbate and hydrogen peroxide (30 mM, 1:1). After reacting for 2 h, the solution was dialyzed using 3600 Da molecular weight cutoff dialysis membranes and precipitated with ethanol [15,31]. UF was obtained using anion-exchange chromatography, as previously described [15]. 

### 4.2. Analytical Methods

The total sugar content of UF was determined according to the method of Dubois et al., using l-fucose as the standard [32]. The sulfate content was analyzed using the barium chloride-gelatin method of Kawai et al. [33]. Uronic acid was estimated via a modified carbazole method, using d-glucuronic acid as the standard [34]. The neutral sugar composition was determined using HPLC chromatography (Shimadzu, LC-20, Kyoto, Japan) [35]. The molecular weight of the sample was assayed by an HP-GPC system at 40 °C, where 2.84% Na_2_SO_4_ solution was used as the mobile phase, with a flow rate of 0.5 mL/min (Shimadzu). A TSK G300 column (300 mm × 7.8 mm) and 2140 refractive index detector were used. A series of dextrans with different molecular weights were purchased from the National Institute for the Control of Pharmaceutical and Biological Products (China) and used as standards. The UF structure was previously studied by our group using Electrospray Mass Spectrometry in Tandem with Collision-Induced Dissociation Tandem Mass Spectrometry (ESI-CID-MS/MS) [21].

### 4.3. Cell Culture and Treatments 

We used a dopaminergic cell line, SH-SY5Y, to establish an in vitro model for study. SH-SY5Y cells were kindly provided by Professor Aiguo Shen (Nantong University) and maintained in Dulbecco’s modified Eagle medium/F12 supplemented with 10% newborn calf serum in an incubator with an atmosphere of 5% CO_2_ at 37 °C. For all experiments, the cells were seeded on 96-well plates, 24-well plates, or 6-well plates, at a density of 1 × 10^5^ cells/mL for 24 h. The cells were divided into 11 groups, treated with DMEM, H_2_O_2_ (100 µM), H_2_O_2_ (100 µM) + UF (100 mM), H_2_O_2_ (100 µM) + UF (500 mM), H_2_O_2_ (100 µM) + UF (800 mM), H_2_O_2_ (100 µM) + MA (100 mM), H_2_O_2_ (100 µM) + LY294002 (20 µM), H_2_O_2_ (100 µM) + UF (100 mM) + LY294002 (20 µM), H_2_O_2_ (100 µM) + UF (500 mM) + LY294002 (20 µM), H_2_O_2_ (100 µM) + UF (800 mM) + LY294002 (20 µM), and H_2_O_2_ (100 µM) + MA (100 mM) + LY294002 (20 µM). Each group had three wells, and each experiment was repeated five times using different batches of cells. 

### 4.4. Measurement of Cell Viability by MTT

The mitochondrial activity was quantitatively assessed using the MTT ([3-(4,5-dimethyl-2-thiazolyl)-2,5-diphenyl tetrazolium bromide]) assay [36]. Briefly, SH-SY5Y cells were plated at a density of 1 × 10^4^ cells/100 µL in 96-well plates. Then, 20 µL of MTT (0.5 mg/mL) reagent was added to each well. After incubating for 4 h at 37 °C, the medium was removed and washed twice with phosphate-buffered solution (pH 7.4); then, 200 µL of DMSO was added to solubilize the formazan crystals. The color that developed was measured at 570 nm using a multiplate reader (Bio-Rad, Hercules, CA, USA). Data were expressed as a percentage of the normal control value. Unless stated otherwise, all other chemicals were purchased from Sigma-Aldrich (St. Louis, MO, USA).

### 4.5. Observation of Morphological Changes

Cells were treated with UF for 24 h. After UF treatment, cells were washed with phosphate-buffered saline and stained with 400 µL of Hoechst 33342 (2.5 µg/mL) for 5 min in the dark. Then, the medium was removed and washed twice with phosphate-buffered solution (pH 7.4) and stained with 400 µL of PI (12.5 µg/mL) for 5 min in the dark. Cells with typical apoptotic nuclear morphology, such as nuclear shrinkage and fragmentation, and micronuclei formation, were identified under fluorescence microscopy and counted using randomly selected fields on numbered slides. The percentage of apoptotic cells was scored by counting at least 200 cells per treatment group, and the average percentage of apoptotic cells was determined for each UF treatment and expressed as the mean ± SD. 

### 4.6. Immunocytochemistry

Immunocytochemistry was performed and modified according to Iida’s study. Briefly, SH-SY5Y cells were washed with PBS three times, fixed with PBS containing 4% (wt/vol) paraformaldehyde for 15 min, and then permeabilized with 0.5% (wt/vol) Triton X-100 in PBS for 20 min. Immunocytostaining was performed with anti-Akt (1:200), anti-P-Akt (1:200), anti-PI3K (1:200), anti-P-PI3K (1:200), anti-BAD (1:200), anti-Bax (1:100), anti-Bcl-2 (1:100), anti-Cytc (1:200), anti-GSK3β (1:200), anti-p53 (1:100), anti-NGF (1:200), and anti-TrkA (1:200) antibodies (Sigma). After the nonspecific reaction was blocked with PBS containing 10% (wt/vol) bovine serum albumin (BSA), the cells were incubated with the primary antibody in PBS overnight, washed with PBST, and incubated with the second antibody (1:200) in PBST for 1 h. After the samples were washed with PBS three times, they were embedded in DAPI for 5 min and then washed with PBST four times. The images were obtained using an Olympus microscope (Shanghai, China). The mean fluorescence intensity was calculated by Image-Pro software (Meyer, TX, USA).

### 4.7. Real-Time PCR

Cells with different treatments were harvested in 350 µL of buffer RL regent (Takara 9767). The total RNA was extracted and quantified, and the integrity was tested using gel electrophoresis. From each sample, 1 µg of total RNA was retrotranscribed into cDNA (Takara RR047A, Dalian, China). Then, 2 µL of each sample was used as a template for amplification reactions conducted with the SYBR Premix Ex Taq^TM^ II (Takara Biotechnology), following the manufacturer’s instructions. The PCR amplifications were conducted using a Life Technology 7500 fast Real-time PCR system (Grand Island, NY, USA). The amplicon for each of the analyzed genes was cloned, and known amounts of the cloned product were used to generate a standard curve using the indicated software. For each sample, duplicate measurements were performed, and the gene copy number was normalized by the amount of glyceraldehyde-3-phosphate dehydrogenase (GAPDH) in the same sample. Primer selection was performed using the Primer Premier Design Software, version 1.0 (Idaho Technology Inc., Alameda, CA, USA). The mRNA level of the control group was set as 100%. Primer sequences are listed in Table 2.

### 4.8. Caspase-3, Caspase-8 and Caspase-9 Activities

After treating cells with UF for 24 h, the cells were harvested using cell scrapers and washed with ice-cold PBS. Then, the cells were lysed for 30 min on ice in 100 µL of cell lysis reagent supplemented with a complete protease inhibitor cocktail. The protein concentration of cell lysates was determined by a Bicinchoninic acid (BCA) assay. The Caspase-3,-8,-9 were tested using a Caspase-3, -8, -9 Activity Assay Kit, respectively, produced by the Beyotime company. The principle of the Caspase -3, -8, -9 activity test is that Caspase-3, -8, -9 could catalyze Ac-DEVD-pNA (acetyl-Asp-Glu-Val-Asp p-nitroanilide), Ac-IETD-pNA(acetyl-Ile-Glu-Thr-Asp p-nitroanilide), and Ac-LEHD-pNA (acetyl-Leu-Glu-His-Asp p-nitroanilide) to produce pNA (p-nitroaniline), respectively. pNA exhibits a strong abosorption at 405 nm.

### 4.9. Western Blotting 

After the H_2_O_2_ or H_2_O_2_ + UF treatment, the cells were harvested using cell scrapers and were washed in ice-cold PBS, and then, they were lysed with two different ice-cold lysis buffers. The supernatants were collected for protein determination using a BCA assay and the protein was run in NuPage Bis-Tris 10% gels and transferred to PVDF membranes. The membranes were blocked in 5% skim milk, 0.05% Tween 20, and Tris-buffered saline (TBS) for 1 h. The PVDF membranes were incubated with primary antibodies: rabbit polyclonal P-PI3K (1:1000), rabbit polyclonal Gsk3b antibody (1:1000), rabbit polyclonal pAkt antibody (1:1000), and rabbit polyclonal GAPDH antibody (1:1000), overnight. The next day, horseradish peroxidase-conjugated secondary antibodies were applied. Peroxidase-conjugated streptavidin and substrate were used for detection. Negative controls were performed by omitting the primary antibodies. The images were analyzed using the NIH Image J software, and the mean optical density of the control group was set as 100%.

### 4.10. Statistical Analysis of Data

The data are presented as the mean values ± SD (*n* = 3–5). The data were analyzed by a one-way ANOVA, a Duncan’s multiple-range test, and an LSD test at a significance level of *p* < 0.05. SPSS 22.0 software was used for the analysis.

## 5. Conclusions

In conclusion, our results clearly demonstrate that UF provide strong neuroprotection against dopaminergic neurodegeneration, partially via the PI3K/Akt pathway. This study indicates that UF may have a “therapeutic antiapoptosis” utility for PD by enhancing the phosphorylation of the PI3K/Akt signaling pathway and impairing caspase-3 levels in H_2_O_2_-induced SH-SY5Y cells. The PI3K/Akt pathway may play an important role in regulating dopaminergic neuronal survival during UF-mediated neuroprotection in PD; the modulation of this PI3K/Akt/caspase-3 pathway may also provide a therapeutic benefit in other CNS diseases. 

## Figures and Tables

**Table 1:** Chemical composition (%, dry weight) and molecular weight of DF and its fraction (UF) isolated from *L. japonica*.

Sample	Fucose	Uronic Acid	Sulfate	Molecular Weight (Da)	Neutral Sugar (Molar Ratio) ^a^ Fuc Gal Man Glc Rha Xyl					
DF	28.7	3.65	30.1	9544	1.000	0.579	0.038	0.159	0.054	0.033
UF	19.12	14.25	21.21	6500	1.000	0.713	0.112	0.257	0.113	0.087

^a^ Gal: galactose; Fuc: fucose; Glc: glucose; Rha: rhamnose; Xyl: xylose; Man: mannose.

**Table 2:** Primers used for RT-PCR.

Target Genes		Primer Sequences	Amplicon (bp)
*Akt*	FP	GCCGCTACTACGCCATGAAGA	214
	RP	CCCGTTGGCATACTCCATCAC	
*Bad*	FP	AGGCCATCAGCAACAACATAAGT	158
	RP	GACAGCTTTGTGCTGGATCTGTG	
*Bax*	FP	GGCGAATTGGACATGAAC	182
	RP	CCGAAGTAGGAGAGGAGG	
*Bcl-2*	FP	CCCCAGAAGAAACTGAACC	195
	RP	GCATCTCCTTGTCTACGC	
*CytC*	FP	TGATGAGGAGATGGCTTG	181
	RP	TCTGTTTCTTTGCGTGGA	
*GSK3β*	FP	ATTCCCTCAAATTAAGGCACCTCC	142
	RP	ATACTCCAGCAGACGGCTACACAG	
*p53*	FP	GGCGAATTGGAGATGAAC	156
	RP	CCGAAGTAGGAGAGGAGG	
*PAkt*	FP	TCTACAACCAGGACCATGAGAA	117
	RP	GAGTAGGAGAACTGGGGAAGT	
*NGF*	FP	TCCAGGTGCATAGCGTAATG	195
	RP	CTCCGGTGAGTCCTGTTGAA	
*TrkA*	FP	ATGAGACCAGCTGTATCT	167
	RP	CATTCTCAAGTGGGAGC	
*GAPDH*	FP	TTCACCACCATGGAGAAGGC	247
	RP	GGCATGGACTGTGGTCATGA	

## Supplementary Materials

The following are available online at www.mdpi.com/1660-3397/15/4/110/s1, Figure S1. The effect of the samples on the neuronal injury induced by H_2_O_2_, Figure S2. Nuclear morphology of H_2_O_2_ and UF treated SH-SY5Y cells for 48 h, Figure S3. Protective effects of UF on H_2_O_2_-induced SH-SY5Y cell of relative density of proteins, Figure S4. Western blotting analysis of PAkt, PPI3K and GSK3β protein on H_2_O_2_-induced SH-SY5Y cell.
