# Supplementary material for: Sulfated Hetero-Polysaccharides Protect SH-SY5Y Cells from H2O2-Induced Apoptosis by Affecting the PI3K/Akt Signaling Pathway
Source: Mar Drugs. 2017 Apr 6;15(4):110. doi: 10.3390/md15040110 (PMC5408256; doi:10.3390/md15040110)
Supplement: Supplementary file 1 [file marinedrugs-15-00110-s001.pdf]

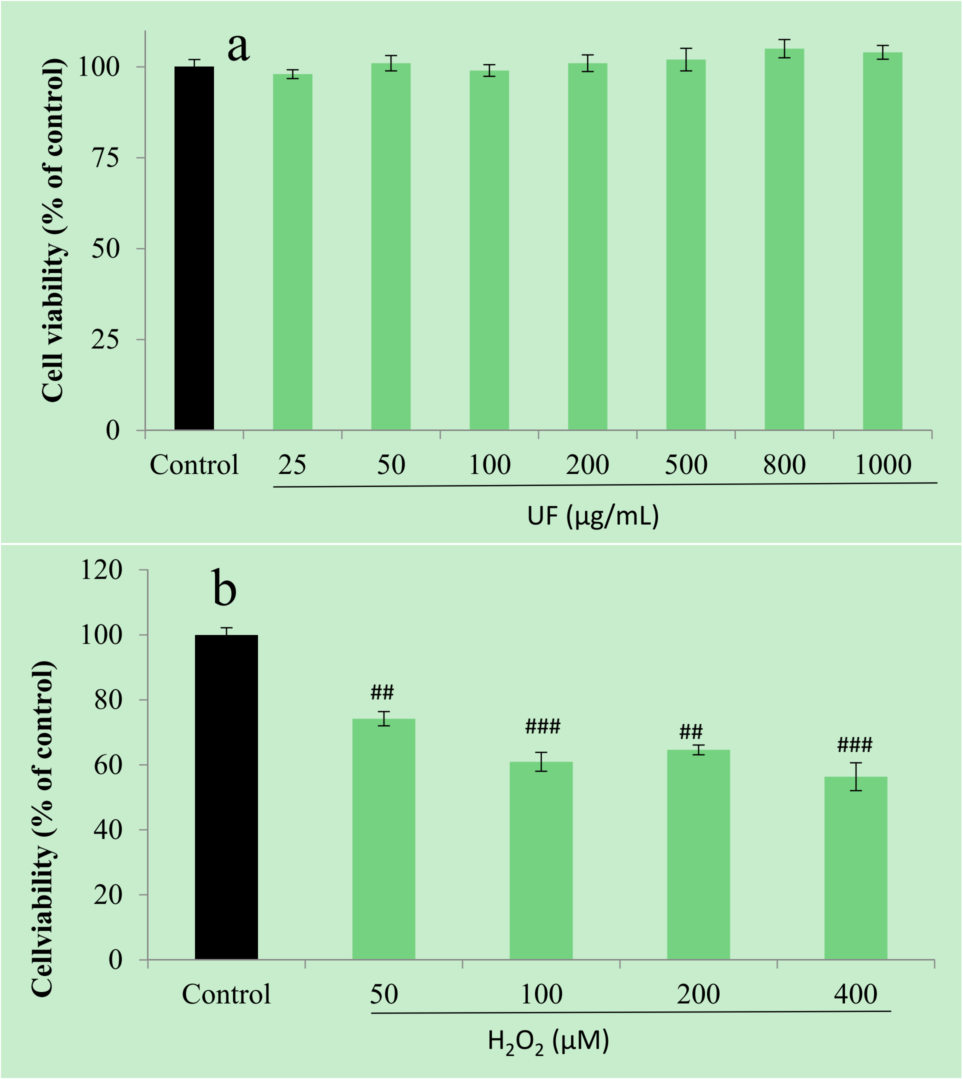

FigureS1. The effect of the samples on the neuronal injury induced by H_2O_2. Effects of UF on SH-SY5Y cell viability (a). Toxic effects of H_2O_2 on SH-SY5Y cell viability (b). ## P<0.01 (Vs control group).

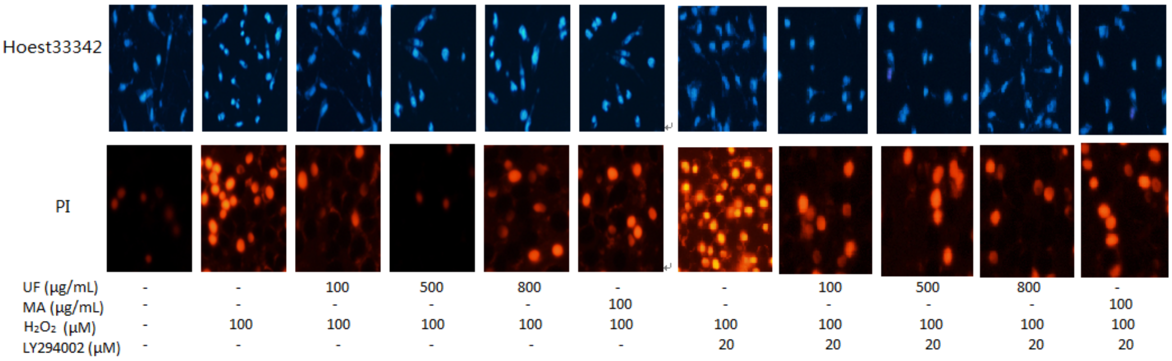

FigureS2. Nuclear morphology of H_2O_2 and UF treated SH-SY5Y cells for 48 h, showing fragmented nuclei and micronuclei after staining with the fluorescent DAN stain Hoechst Bisbenzimide 33342 and observed by fluorescence. The bar scale in the picture is 50 in length.

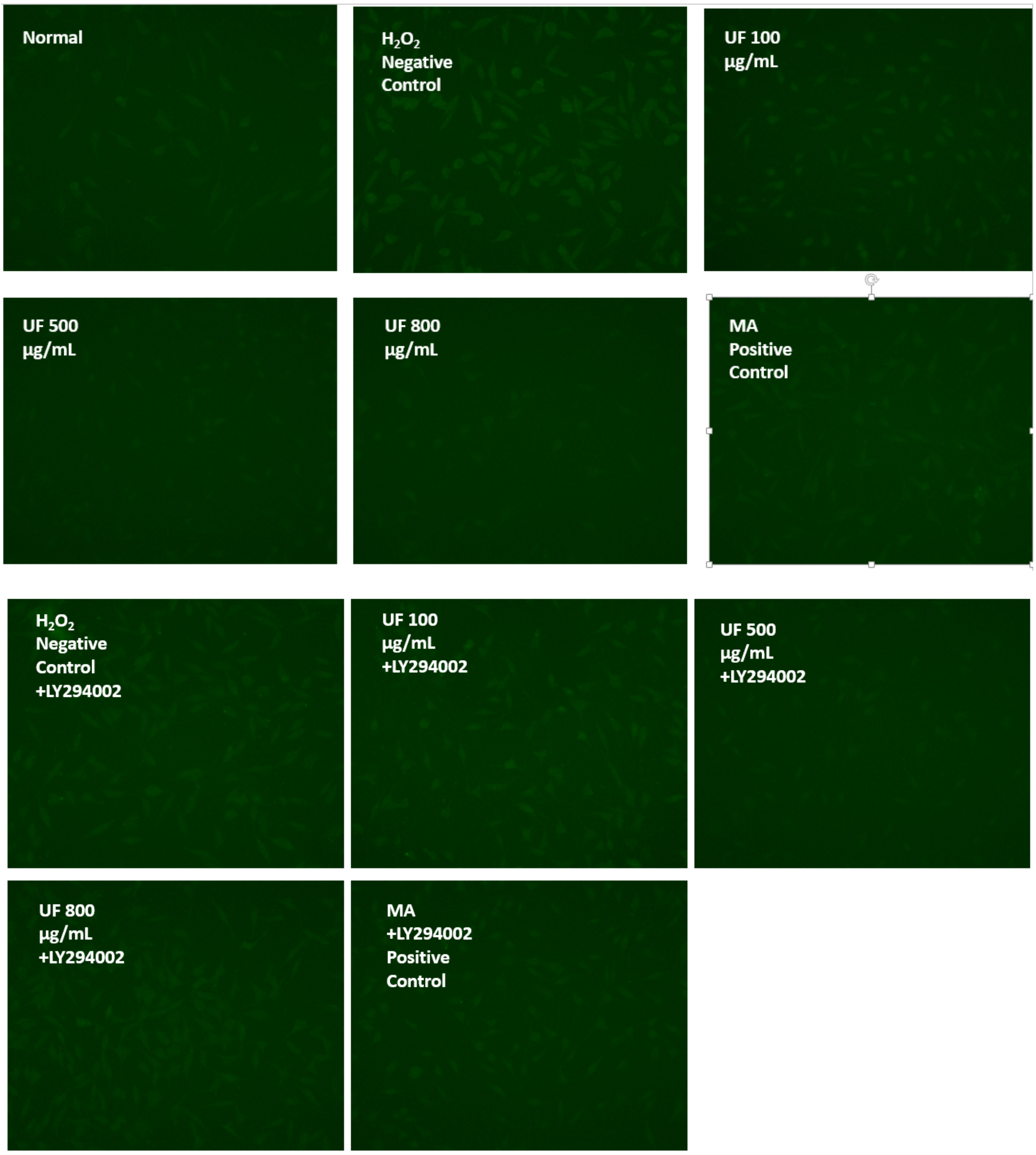

Figure S3a. Protective effects of UF on H_2O_2-induced SH-SY5Y cells of a relative density of AKt protein.

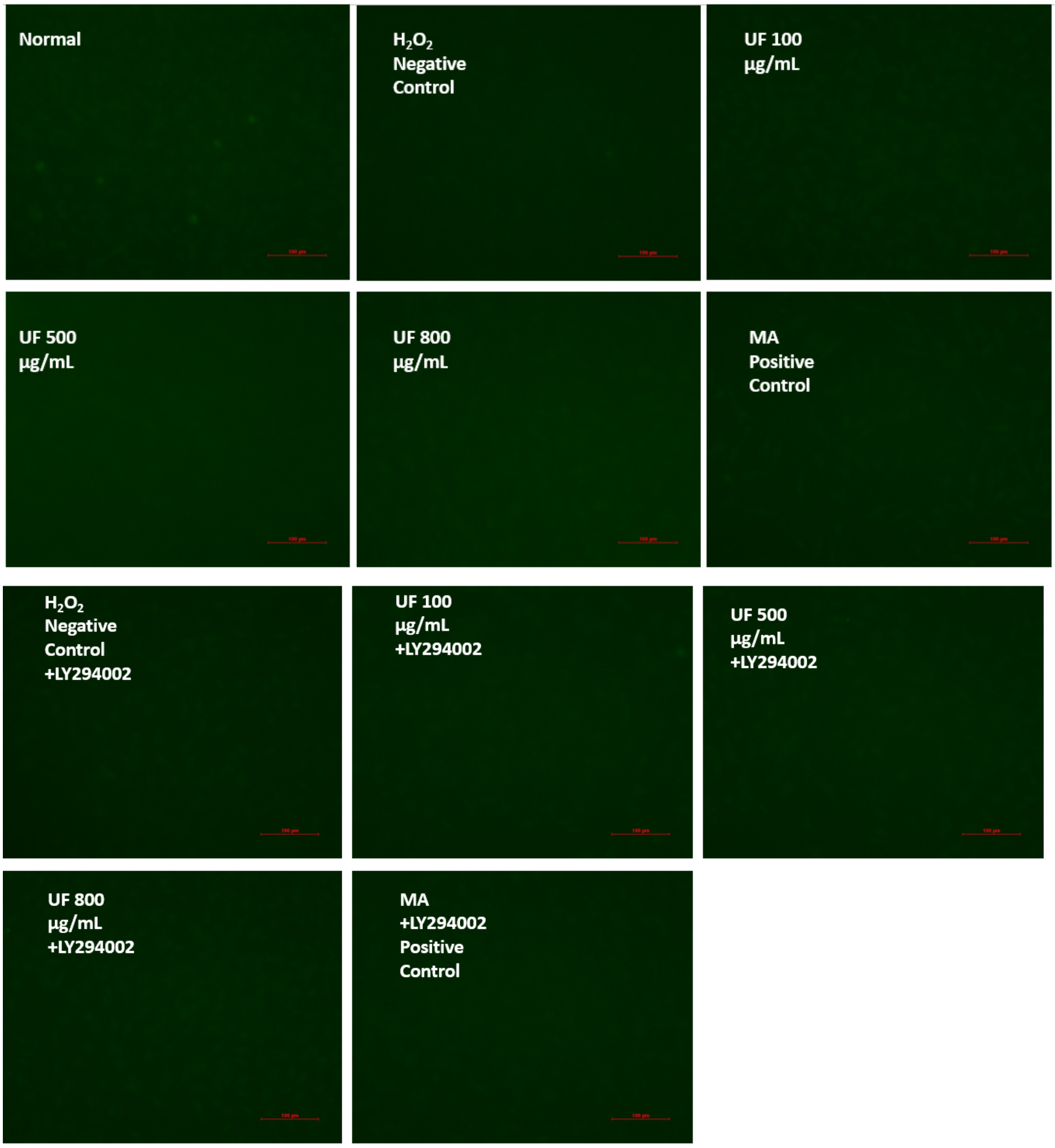

Figure S3b. Protective effects of UF on H_2O_2-induced SH-SY5Y cells of a relative density of PAKt protein.

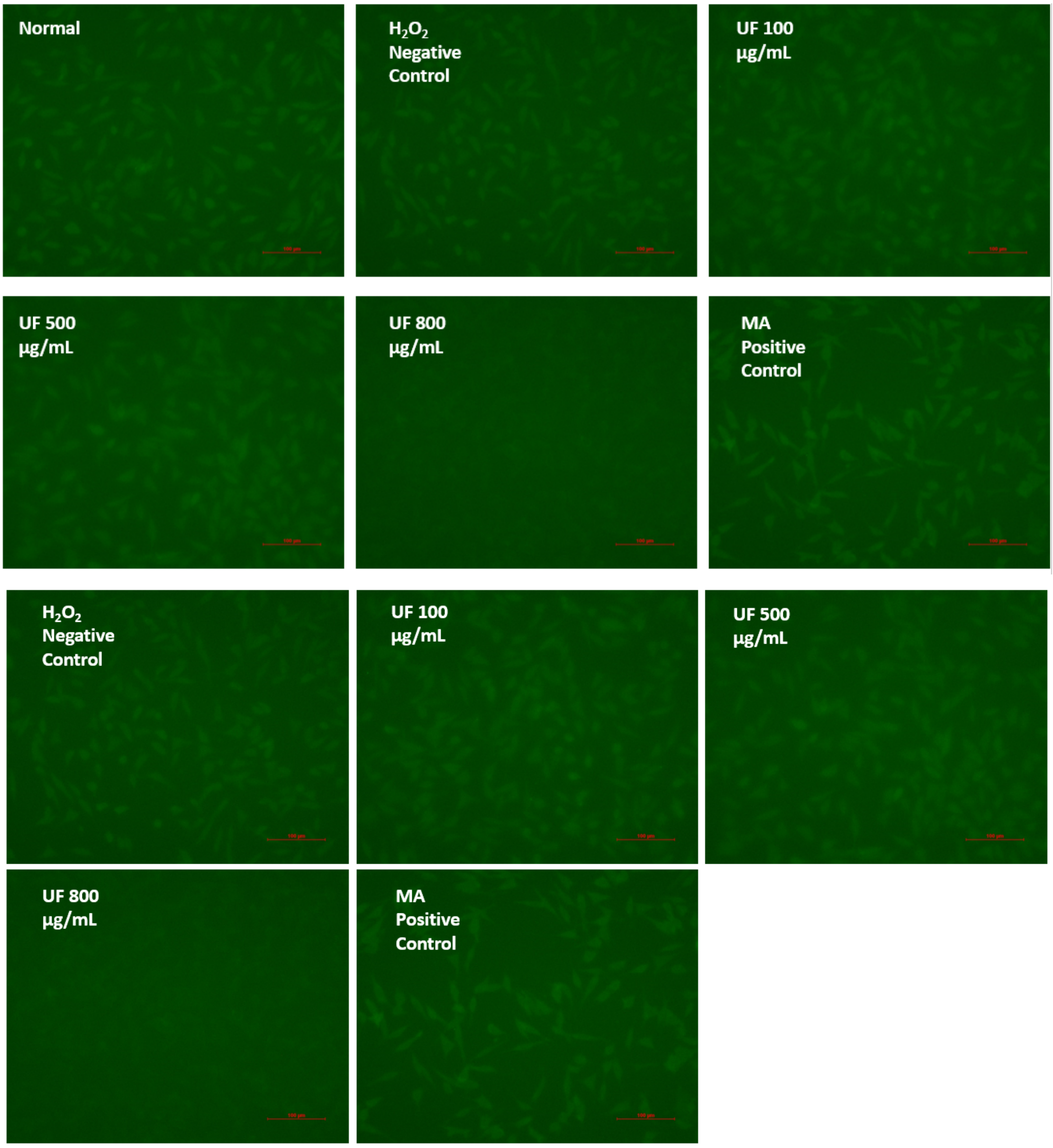

Figure S3 c. Protective effects of UF on H_2O_2-induced SH-SY5Y cells of a relative density of PI3K protein.

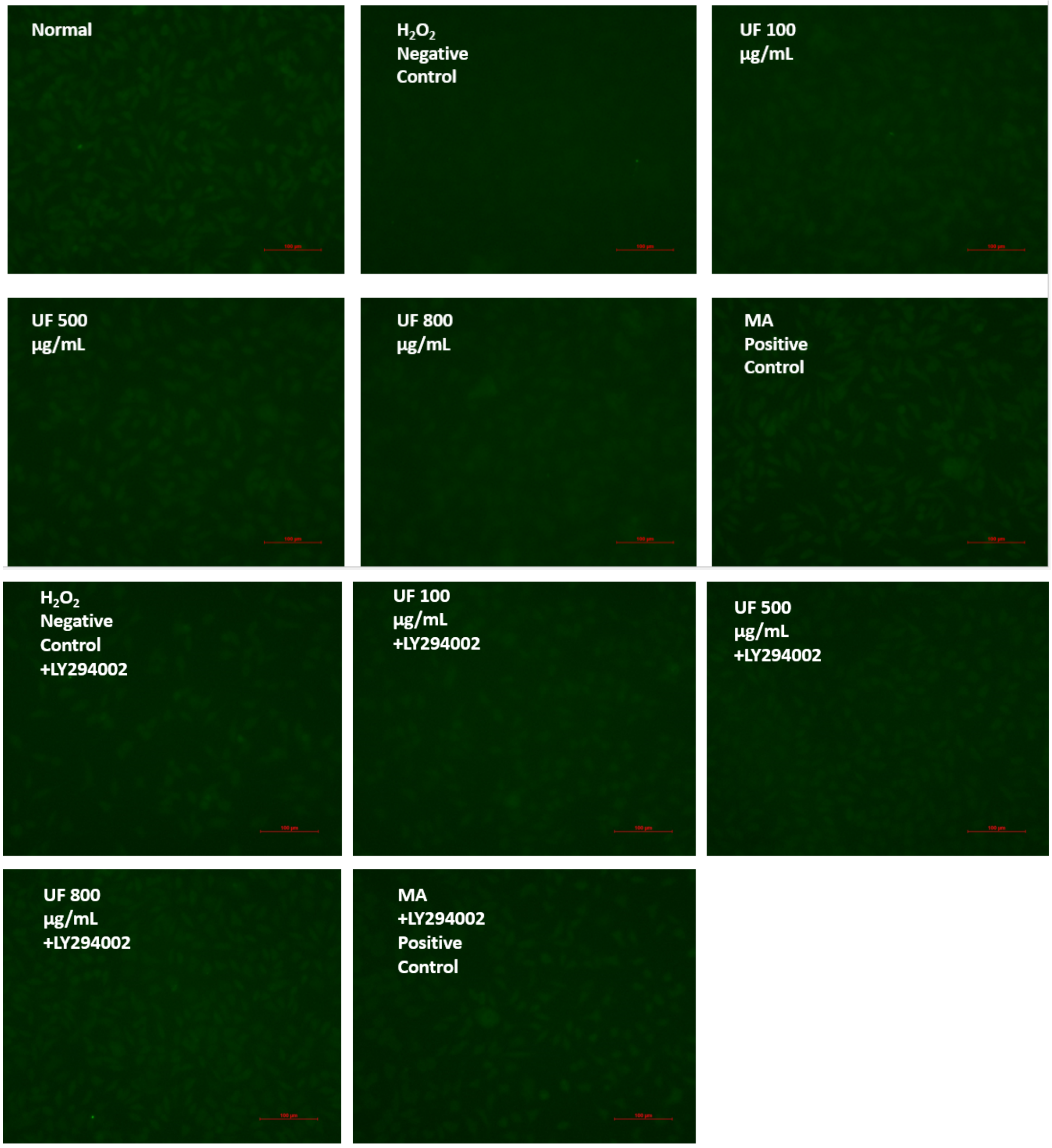

Figure S3 d. Protective effects of UF on H_2O_2-induced SH-SY5Y cells of a relative density of P-PI3K protein.

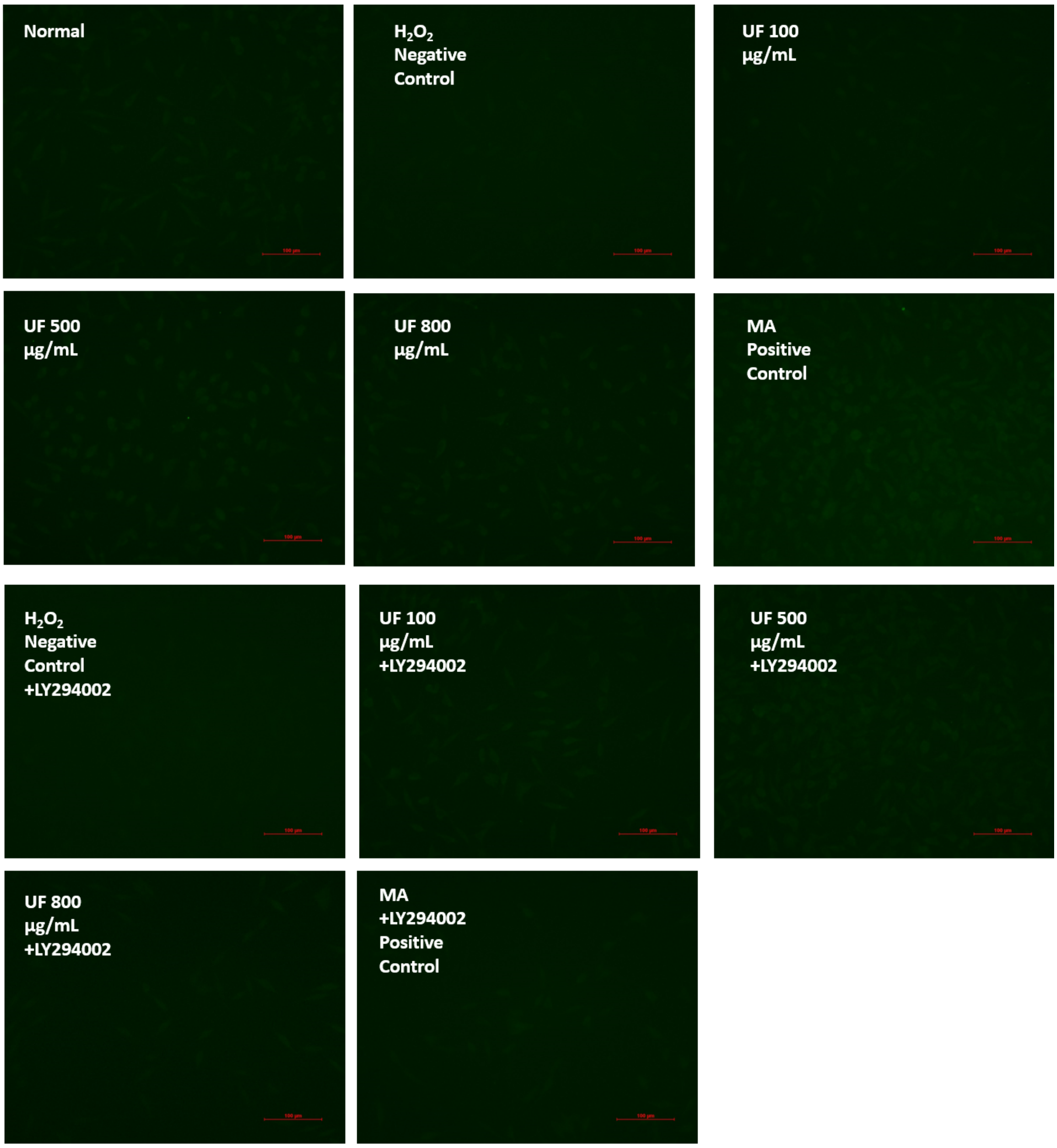

Figure S3 e. Protective effects of UF on H_2O_2-induced SH-SY5Y cells of a relative density of Bcl-2 protein.

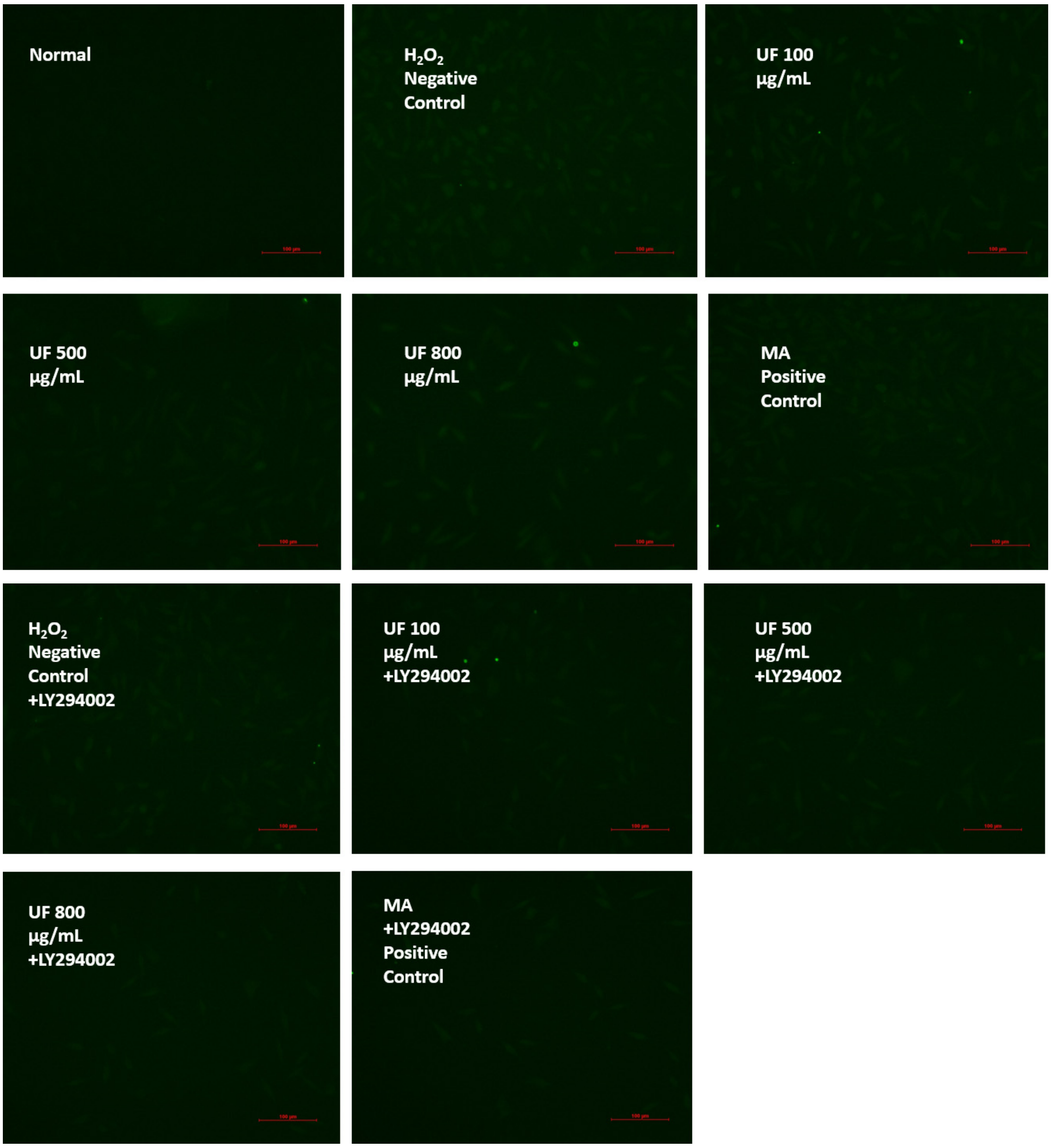

Figure S3 f. Protective effects of UF on H_2O_2-induced SH-SY5Y cells of a relative density of BAD protein.

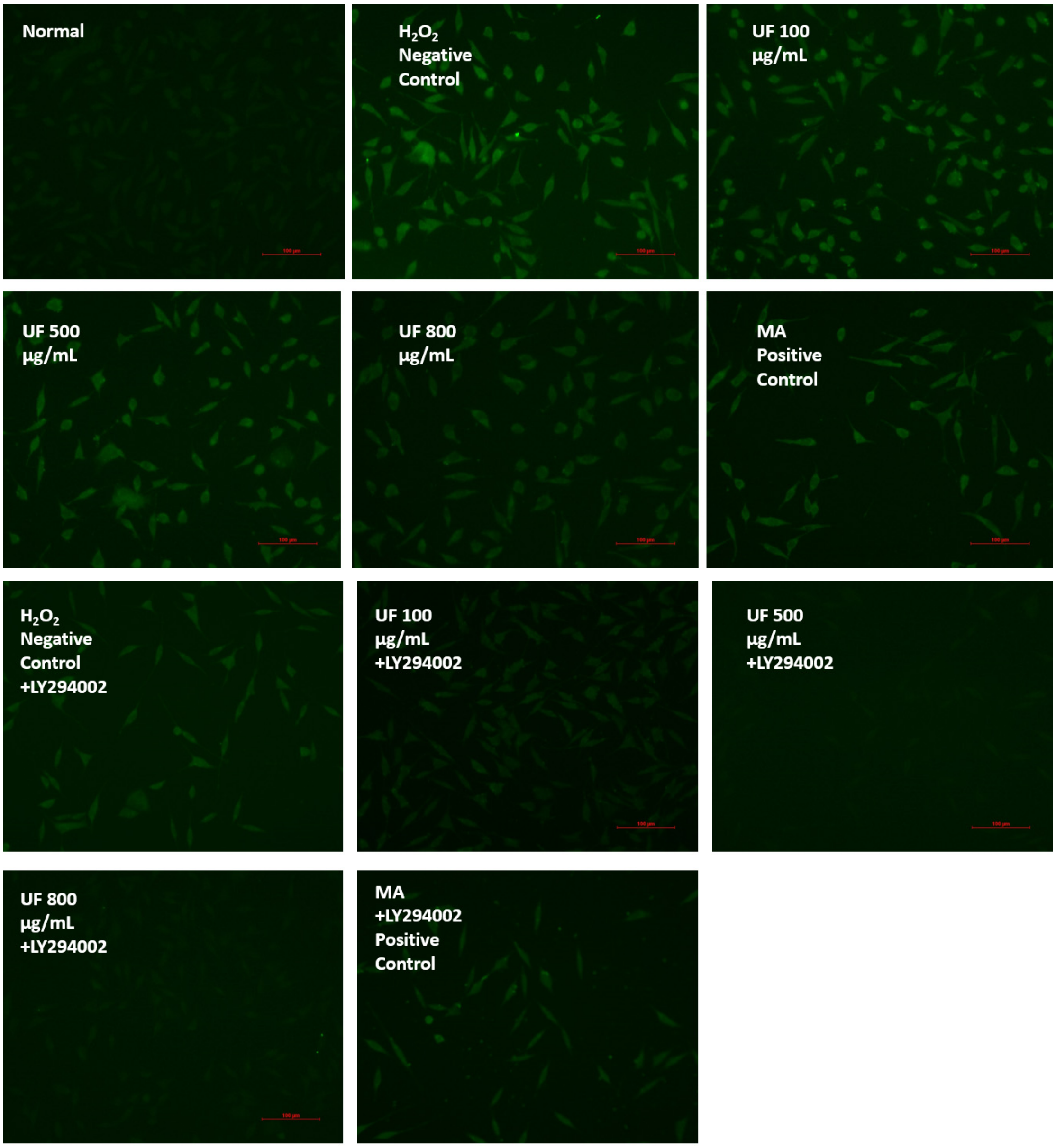

Figure S3 g. Protective effects of UF on H_2O_2-induced SH-SY5Y cells of a relative density of Bax protein.

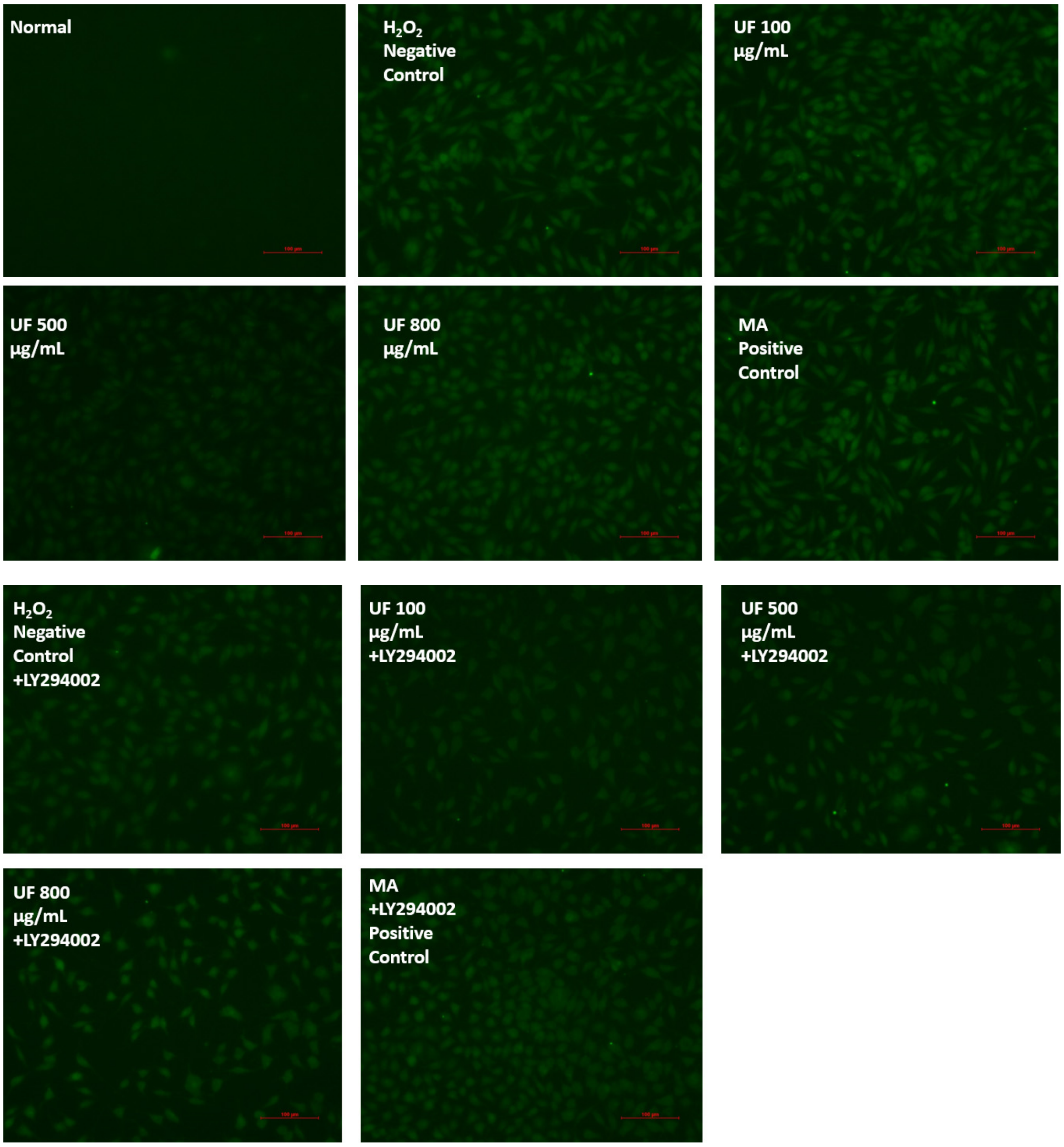

Figure S3 h. Protective effects of UF on H_2O_2-induced SH-SY5Y cells of a relative density of p53 protein.

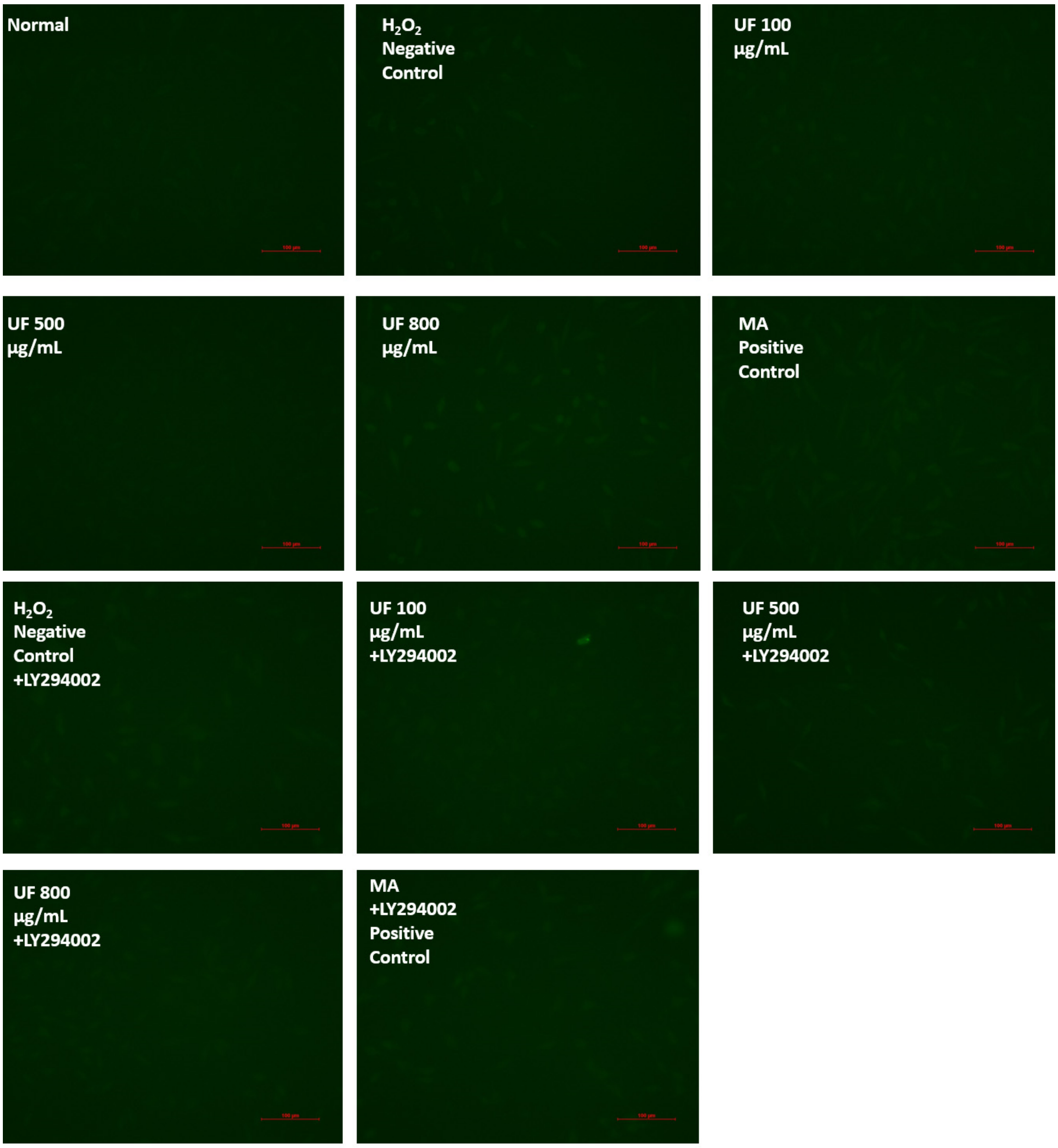

Figure S3 i. Protective effects of UF on H_2O_2-induced SH-SY5Y cells of a relative density of Cytc protein.

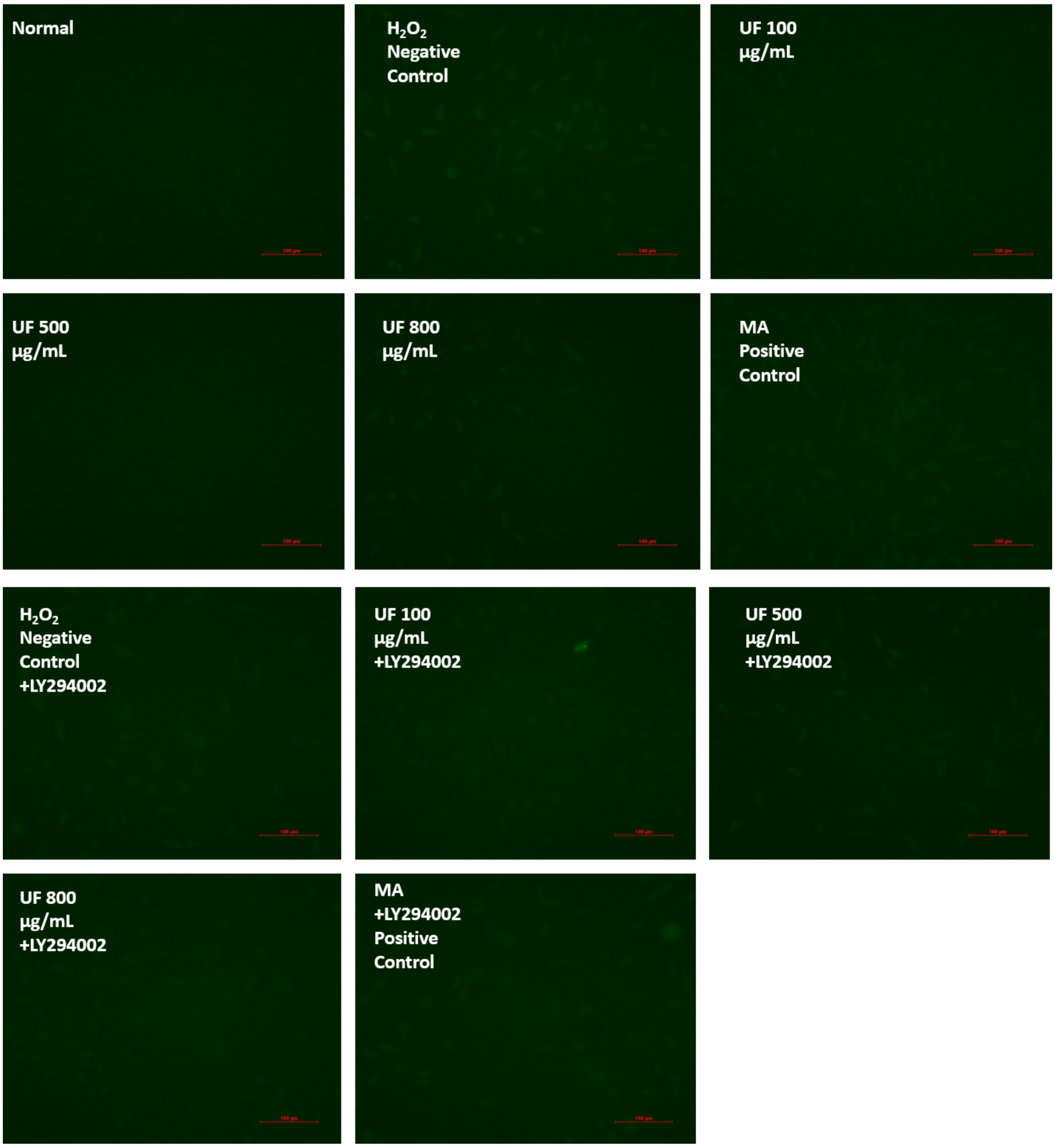

Figure S3 j. Protective effects of UF on H_2O_2-induced SH-SY5Y cells of a relative density of GSK3β protein.

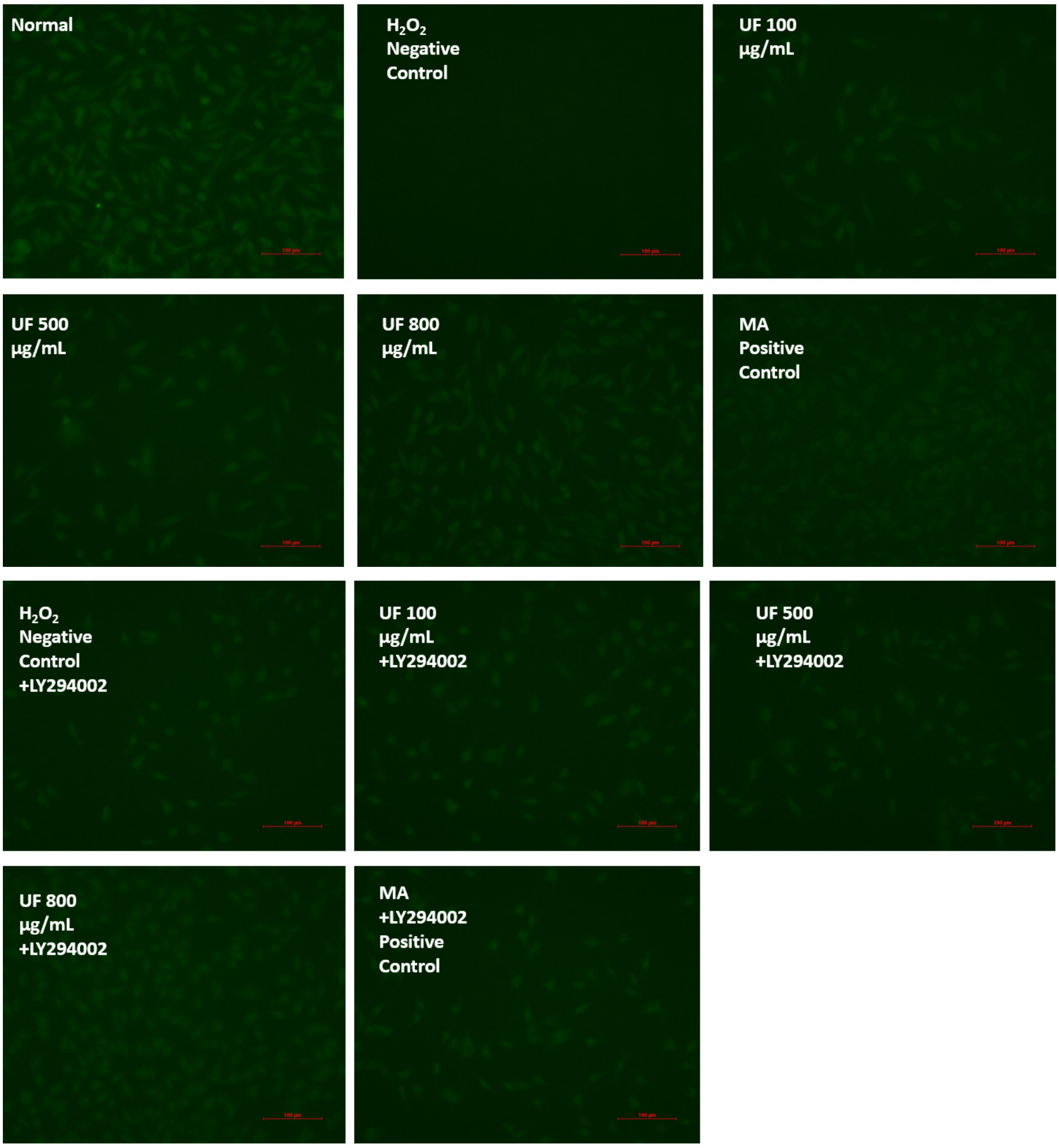

Figure S3 k. Protective effects of UF on H_2O_2-induced SH-SY5Y cells of a relative density of NGF protein.

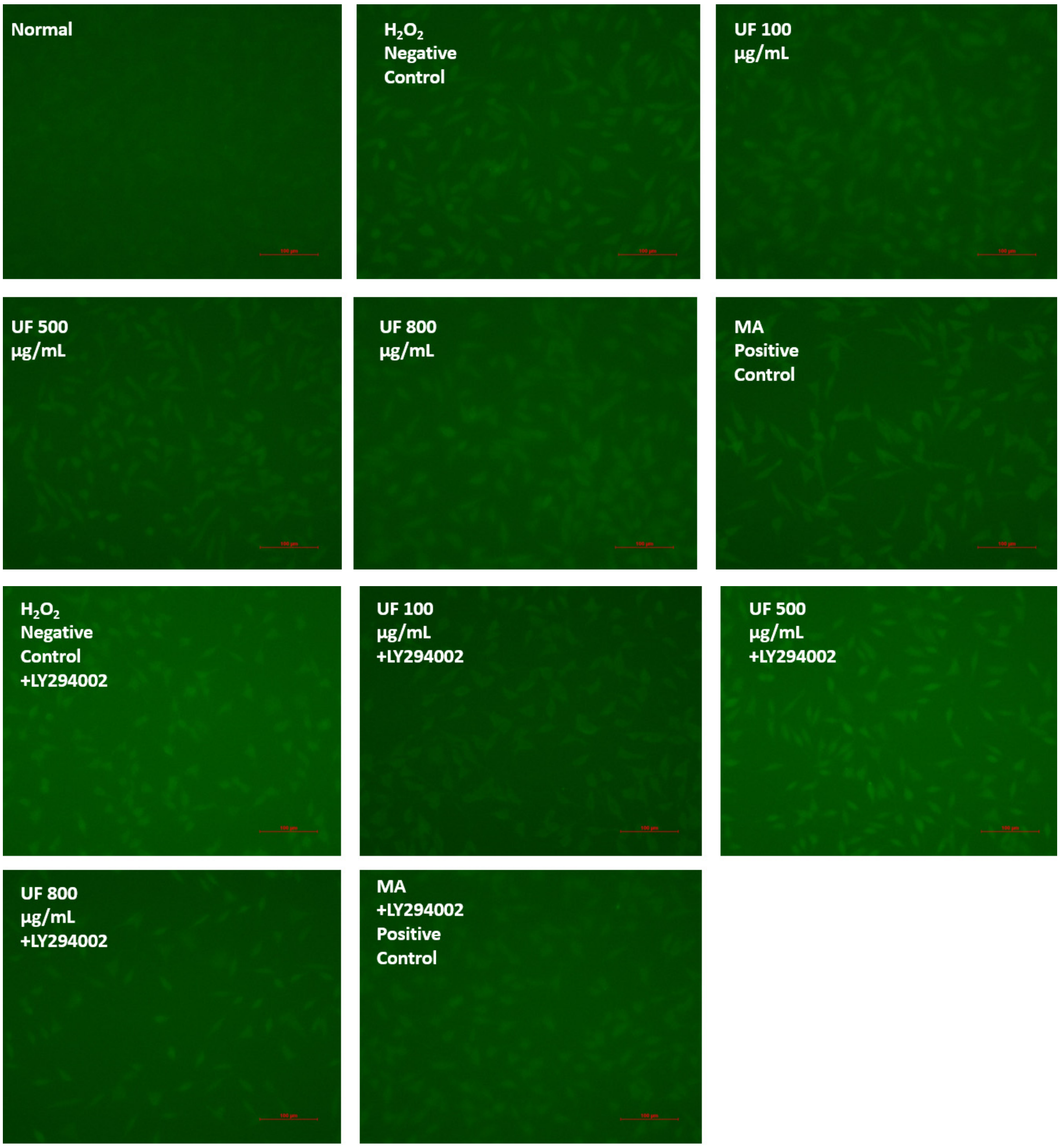

Figure S3 l. Protective effects of UF on H_2O_2-induced SH-SY5Y cells of a relative density of TrkA protein.

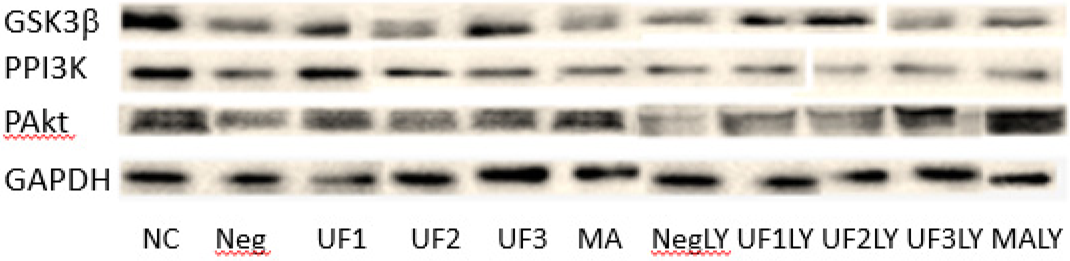

Figure S4. Western blotting analysis of PAkt, PPI3K, and GSK3β proteins on H_2O_2-induced SH-SY5Y cells. NC: Normal Control group, Neg: Negtive Control group, UF1: UF 100 μg/mL group, UF2: UF 500 μg/mL group, UF3: UF 800 μg/mL group, MA: Positive Control group, NCLY: Normal Control group+LY294002, NegLY: Negtive Control group+LY294002, UF1LY: UF 100 μg/mL group+LY294002, UF2LY: UF 500 μg/mL group+LY294002, UF3LY: UF 800 μg/mL group+LY294002, MALY: Positive Control group+LY294002, $^{\#}P<0.05$, $^{\#\#}P<0.01$, $^{\#\#\#}P<0.001$ (Vs NC), * P<0.05, ** P<0.01, *** P<0.001 (Vs Neg), ^P<0.05, ^^P<0.01, ^^^P<0.001, (Vs NegLY).
